# Arterial spin labeling provides a reliable neurobiological marker of autism spectrum disorder

**DOI:** 10.1186/s11689-018-9250-0

**Published:** 2018-12-13

**Authors:** Benjamin E. Yerys, John D. Herrington, Gregory K. Bartley, Hua-Shan Liu, John A. Detre, Robert T. Schultz

**Affiliations:** 1 Center for Autism Research, The Children’s Hospital of Philadelphia, Roberts Center for Pediatric Research, 2716 South Street, 5th floor, Philadelphia, PA 19146-2305 USA; 2 Department of Psychiatry, University of Pennsylvania, 3400 Civic Center Boulevard, Philadelphia, PA 19104 USA; 3 Department of Neurology, University of Pennsylvania, 3400 Civic Center Boulevard, Philadelphia, PA 19104 USA; 4 Department of Pediatrics, Perelman School Medicine, University of Pennsylvania, 3400 Civic Center Boulevard, Philadelphia, PA 19104 USA

**Keywords:** Social cognition, Social perception, MRI, Autism, Faces, Blood flow

## Abstract

**Background:**

Research on neurobiological markers of autism spectrum disorder (ASD) has been elusive. However, radionuclide studies of cerebral blood flow (CBF) have shown decreased blood flow (hypoperfusion) in the temporal lobes of individuals with ASD across ages and intelligence. This observation fits with current neuroscientific models that implicate temporal regions in social perception and social cognition. Arterial spin labeled perfusion MRI allows noninvasive quantification of regional CBF as part of a multimodal MRI protocol. This method is almost entirely absent from ASD research to date. Our a priori hypothesis was that children with ASD would present with hypoperfusion in the temporal lobes—most notably the fusiform gyrus (given its prominent role in ASD social perception deficits). We also sought to examine the reproducibility of CBF measures, and their relationship to individual differences in facial recognition and ASD symptoms.

**Methods:**

A total of 58 males (33 with ASD) between the ages of 12 and 17 years participated in the study. All children completed two arterial spin labeling and structural (T1) scans using a 3 T Siemens Verio scanner approximately 8 weeks apart, as well as behavioral testing at time 1 that included diagnostic measures and the Benton Facial Recognition Test. CBF was the key dependent variable, as was facial recognition performance, and ASD symptoms. The two scans were used for reliability analyses.

**Results:**

The ASD group showed hypoperfusion in the bilateral fusiform gyrus and in right inferior temporal gyrus. Intra-class correlations showed moderate to good reliability across time within both groups, and no diagnostic group × time interactions. CBF in the left fusiform gyrus was significantly positively correlated with facial recognition. No significant correlations were observed with core ASD symptoms.

**Conclusions:**

Arterial spin labeling revealed hypoperfusion in children with ASD in regions critical to social perception and cognition. The left fusiform gyrus plays an important role in facial recognition, and greater CBF in this region was correlated with more normative facial recognition performance in children with ASD. This study takes an important first step in establishing CBF of the temporal lobes as a reliable marker of ASD.

## Background

Research into the neurobiology of autism spectrum disorders (ASDs) is marked by limitations in the ability to identify reliable, biologically based markers that can confirm diagnosis. However, nearly all perfusion imaging studies in individuals with ASD report decreased cerebral blood flow (CBF) to the temporal cortices [1–8], but see [9] for counterevidence. These results are surprisingly consistent, considering many of these studies differed greatly on key variables: phenotypic classification of ASDs, sample characteristics, scanning task, and CBF scanning parameters. Results to date suggest that hypoperfusion observed in the temporal lobes of individuals with ASD may be a viable marker that spans age and intellectual level in the identification of ASD. However, no study has tested the reliability of hypoperfusion in the temporal lobes of individuals with ASD over time, and the relationship of hypoperfusion to the behavioral phenotype of ASD remains underspecified.

CBF changes are thought to reflect regional changes in neural activity in a region over time [10, 11]. Furthermore, CBF provides absolute quantification of regional brain function. It may therefore provide a more reliable marker of trait-like effects than measures obtained through condition contrasts, such as blood-oxygen-level-dependent scanning that is typically used in task-based functional magnetic resonance imaging (MRI) [12–18].

Arterial spin labeled (ASL) perfusion MRI utilizes magnetic labeling of endogenous blood water as a tracer for CBF quantification [19]. This non-invasive approach for CBF mapping eliminates the risk of using radioligand tracers with pediatric populations—as have been used in nearly all CBF studies with ASD samples to date. ASL can readily be combined with other MRI modalities. ASL is also particularly sensitive in younger populations, where the increased water and blood flow rates in the brains of children allow for SNR improvements of over 70% relative to adults [20–22]. As a result, ASL has seen increased use in the pediatric prevention, identification, and treatment of neurodevelopmental disorders such as periventricular leukomalacia, ischemic stroke, and sickle-cell-related cerebral ischemia [22]. Despite these strengths, ASL has rarely been used to examine CBF differences in ASD.

In functional imaging applications, ASL can be used to detect changes in CBF occurring over several minutes or longer [23, 24]. It can also be used to measure task-specific changes in CBF (analogous to traditional fMRI). For example, perfusion fMRI based on ASL was used to demonstrate activity in the temporal lobe during passive viewing of a video [25], and to accentuate differences in mild cognitive impairment, another disorder frequently associated with temporal lobe function [26]. An ability to measure brain function during a sustained task condition such as passive viewing of a video facilitates implementation in both a clinical setting, and with a broader range of cognitive functioning for individuals with ASD. Furthermore, watching a movie in a relaxed state may also be as close to the context of “natural vision” as can be achieved during an MRI scan. Studies incorporating movies, comics, stories, and other contextually centered stimuli are known to elicit greater temporal pole activation with functional magnetic resonance imaging [27], and resting state fMRI has revealed that the temporal pole has strong functional connectivity with brain areas highly relevant to the processing of social semantic detail and the elicitation of visceral emotional responses [28]. Impaired function of these networks would have implications for social functioning and for disorders like ASD that are characterized by atypical social functioning.

Here, we utilized ASL MRI to compare temporal lobe function during passive video viewing in a cohort of children with ASD to typically developing control (TDC) participants. Although it is likely that ASD is associated with differences in function that are distributed across the brain, we focused the present paper primarily on the temporal lobes, as difference in visual association areas are among the most widely replicated in ASD [29–33]. We hypothesized that regional CBF in the temporal lobe would be reduced in children with ASD as compared to controls, reflecting reduced activity in visual association areas (fusiform gyrus, posterior superior temporal sulcus, temporal pole) and language areas (Wernicke’s area) that have been linked to social cognition and language impairments in ASD [29, 34, 35]. This hypothesis is based on the vast majority of prior perfusion studies highlighting hypoperfusion in the temporal lobe [1–8], meta-analyses of task-based functional MRI using blood-oxygenation-level-dependent response implicating the fusiform gyrus in social cognition [30, 31], and a recent large-scale international study highlighting atypical structural development of the temporal lobes [36]. We also sought to assess the reproducibility of ASL over the time interval of a typical clinical trial of an investigational drug, i.e., about 2 months, so that ASL might prove to be a reliable biomarker that could be easy to implement as an outcome measure (i.e., resting/no onerous task demands) for future treatment trials. Moreover, we sought to better understand blood perfusion in key brain regions by comparing changes in ASD versus TDC measured by ASL during passive video viewing. Furthermore, given the important role of the fusiform gyrus in processing of faces [29, 37], we explored whether perfusion in this area of the temporal lobe was sensitive to individual differences in social function and processing of faces. Alongside our a priori temporal lobe hypotheses, we conducted whole-brain tests for significant areas of hypoperfusion, and tests of global differences in CBF.

## Methods

### Participants

Seventy-six children (43 ASDs; 33 TDCs) between the ages of 12 and 17 were invited to participate in this study. To be included in the ASD group, children needed a community diagnosis of autism, Asperger’s syndrome, or pervasive developmental disorder; this study conducted data collection prior to the release of DSM-5 [38] so that is why DSM-IV-TR [39] criteria were used. This diagnosis was confirmed by expert, research reliable clinicians who used the revised algorithm for the Autism Diagnostic Observation Schedule [40] and the Autism Diagnostic Interview-Revised [41] to inform their diagnostic decision with a DSM-IV-TR checklist. Children were not invited to participate or excluded if they failed to meet criteria for an ASD diagnosis based on expert opinion, had active severe psychiatric symptoms that limited participation (e.g., psychosis, severe depression, mania), parents reported a known genetic disorder (e.g., fragile X), or severe premature birth (< 32 weeks). Nine eligible children withdrew from the study prior to the imaging phase, six were excluded from the current analyses due to poor data quality, and one was removed due to a scanner error. Because ASL data were successfully collected on only two female ASD participants, the analyzed sample was restricted to males, for a final sample of 58 participants (33 ASD, 25 TDC). TDCs were screened and excluded if parents reported any known genetic, language, learning, neurological, or psychiatric disorder, premature birth, any first- or second-degree relative with ASD, or receiving any psychoactive medication. TDCs were also excluded if they presented with elevated symptoms on the parent-report Child and Adolescent Symptom Inventory-Revised [42].

### General study procedures

All participants completed three data collection sessions. The first was a behavioral evaluation to confirm children met inclusion and exclusion criteria, to assess cognitive ability, and to characterize participants along broad spectrum of ASD behavior. Measures including the Differential Ability Scales, second edition (DAS-II; [43]), Social Responsiveness Scale-second edition (SRS; [44]) the Vineland Adaptive Behavior Scales-II (VABS-II; [45]), and the Benton Facial Recognition Task [46] (see Table 1 for group characteristics). All participants then completed two scan sessions approximately 8 weeks apart to assess scan-rescan reliability (mean difference in days ASD = 8.87 ± 0.73, TDC = 8.97 ± 0.65).

**Table 1:** Participant characteristics

	ASD *n* = 33	TDC *n* = 25	*p* value	Cohen’s *d*^a^
Age (years)–M(SD)	14.9 (1.73)	14.9 (1.66)	0.98	0.01
FSIQ–M(SD)	94 (20)	123 (18)	< 0.001	1.53
Range	47–133	87–155		
Verbal IQ–M(SD)	99 (22)	124 (20	< 0.001	1.19
Range	48–148	86–165		
Nonverbal IQ–M(SD)	94 (20)	116 (17)	< 0.001	1.18
Range	31–127	92–147		
VABS-II communication	75 (14)	106 (12)	< 0.001	2.37
VABS-II socialization	67 (12)	108 (10)	< 0.001	3.70
SRS total *t* score	77 (11)	44 (5)	< 0.001	3.73
BFRT overall score	40 (4)	42 (5)	0.026	0.59
ADOS social affect	11 (4)	–	–	
ADOS repetitive behaviors	3 (2)	–	–	
ADOS total score	14 (4.00)	–	–	

*ADOS-2* Autism Diagnostic Observation Schedule (revised algorithm to align with second edition scoring), *ASD* autism spectrum disorder, *FSIQ* full scale IQ, *BFRT* Benton Facial Recognition Test, *TDC* typically developing control, *VABS-II* Vineland Adaptive Behavior Scales, second edition

^a^NB: For ease of displaying behavioral data, whole numbers are given, but Cohen’s *d* was calculated on values with two decimal points

### MRI scanning

A Siemens Verio 3 T scanner with a 32-channel head coil was used for scanning. High-resolution structural MRI data (MPRAGE sequence, .9 × .8 × .8 mm, TR/TE = 2000/3.3 ms) were collected for each participant in order to identify brain regions of interest (ROIs) and register data into standard space. Regional CBF (mL/100 g/min) was measured using pseudocontinuous ASL with 2D gradient-echo echo-planar imaging [47]. The labeling and control RF duration was 1.5 s with post-labeling delay of 1.2 s. Multi-slice perfusion maps with 40 label/control pairs were acquired with TR/TE = 4000/17 ms, flip angle = 90^0^, bandwidth = 3005 Hz/pixel, slice thickness = 5 mm, matrix size = 64 × 64, FOV = 220 × 220 mm, and number of slices = 20.

### ASL task procedures

Prior to MRI, participants were desensitized and familiarized with all procedures, and trained in a mock scanner to stay still within 2 mm. All children passively viewed a Discovery Channel video (“Planet Earth: Pole-to-Pole”) without sound on a projection screen during mock scanning, as well as during ASL acquisition at both data acquisition time points. Thus, by time point 2, participants had seen the film three times. The video was initiated at the exact same time point for every subject across all exposures. It intermixed displays of nature scenes (mountains, tundra, clouds, waterfalls, satellite views of earth) 33% of the time, animals in nature scenes (flock of birds, penguins, polar bears) for 60% of the time, and 3 different segments of simple text phrases (“Planet Earth,” “From Pole to Pole,” and “Narrator David Attenborough”) for 7% of the time. During the video, participants were instructed to quietly attend throughout with their eyes open, while a research assistant monitored this at their side in the MRI room. ASL scanning lasted approximately 6 min.

### Image analysis

Raw ASL images were motion-corrected in FMRIB Software Library (FSL; [48]) using a 6-parameter rigid body spatial transformation and co-registered to the MPRAGE images of the same session. ASL averaged difference images were converted to mL/100 g/min using a single-compartment model in the perfusion data processing toolbox, ASLtbx [49, 50]. Structural images were segmented into probabilistic gray matter (GM), white matter (WM), and cerebrospinal fluid (CSF) maps using FMRIB’s Automated Segmentation Tool (FAST), and probabilistic averages of GM and WM designations were converted to binary masks (thresholded at 0.5). These binary masks were combined to create a global mask. The global mask was used as an ROI to extract CBF for whole-brain area from the individual CBF map. ASL data were smoothed at 8 mm full width at half maximum. A binary global mask was then created by adding together GM and WM masks. ASL relative CBF (rCBF) maps were generated by normalizing CBF to global CBF for each subject. In higher-level analysis, affine (12 DOF) transformations to a 2 mm MNI152 template were performed using FMRIB’s Linear Image Registration Tool [51].

### Statistical analysis plan

To examine group differences in ASL, higher-level group contrasts across the two time points were analyzed in the context of a two-way ANOVA examining the effects of time point (repeated measure) and group. FSL’s Threshold Free Cluster Enhancement (TFCE) was performed [48, 52, 53] for within-method differences and when examining convergence between them. TFCE uses a permuted null-distribution of the max statistic to enhance cluster-like structures without subjecting them to binarization, allowing the data to retain voxel-wise relevance. A 10,000 Monte Carlo permutation test with FDR correction was performed on all univariate analyses, which equates to a confidence limit of ± 0.0044 for the chosen significance alpha = 0.05 [54]. To reduce the high spatial frequency noise often introduced through poor standard deviation estimates obtained in smaller sample sizes, variance smoothing of slope estimates was also performed at 5 mm half width at half maximum [55]. Multiple comparisons correction was limited to a sample-averaged temporal lobe gray matter mask (using segmentation from FSL FAST [56]), thresholded at 0.5. As part of a secondary analysis to ensure significant findings were not the result of an IQ outlier, we re-tested our primary findings while excluding the one child with ASD who had an IQ of 47. Also, to confirm significant findings were not missed due to our a priori focus on the temporal lobe, we also examined group differences in rCBF and absolute CBF across the entire brain, as well as a frontal lobe gray matter mask analysis based on prior findings [9]. The frontal lobe gray matter mask was created and thresholded in the same manner as the temporal lobe mask.

In order to assess the scan-re-scan reliability of the rCBF measures within each group, intra-class correlations (ICC) were estimated across the two sessions using a mask for the frontal, insula, occipital, parietal, and temporal lobes. For each mask, we multiplied a standard anatomical mask from the MNI atlas with a sample-averaged gray matter mask that was generated by segmenting the T1 scan with FSL’s FAST and thresholding it at 0.5. Each ICC was a two-way ANOVA mixed model with consistency agreement (Shrout and Fliess’s ICC(3,1) model); these ICCs were calculated using custom scripts in R [57] incorporating the ‘irr’ [58] package.

To explore relationships with ASD symptoms and facial recognition, Pearson product-moment correlation and simple linear regression were used to relate rCBF and various behavioral variables only within the ASD group. Controls were not included in these analyses as their face recognition scores and parent ratings had limited variance (ceiling effects). All significance values were subjected to false discovery rate multiple comparisons correction (alpha = 0.05); 95% confidence intervals were calculated where appropriate.

## Results

### Significant group differences in participant characteristics and behavioral measures

ASD and control groups were matched on age, but not IQ (see Table 1). As predicted, groups differed significantly in parent ratings of communication and socialization adaptive behaviors on the VABS-II, ASD traits on the SRS-2, as well as the Benton Facial Recognition Test.

### Significant temporal lobe group differences in pCASL

**Fig. 1:**
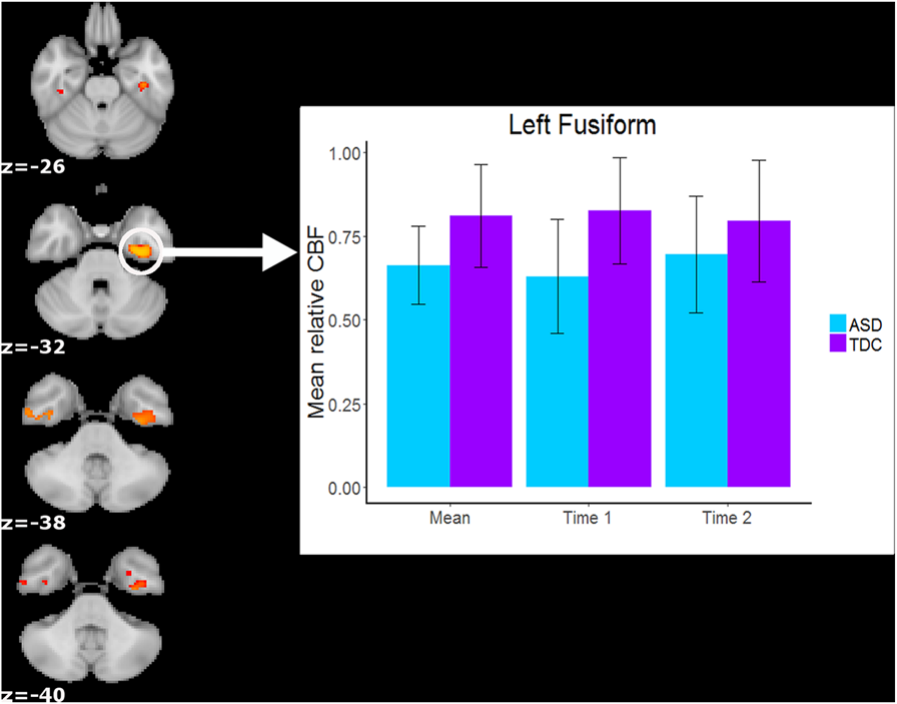
TDC > ASD regional cerebral blood flow (rCBF; *p* < 0.05 corrected with FSL’s threshold-free cluster enhancement algorithm within randomize). Coordinates are in MNI space and are rendered in radiological orientation (right side of image is the left hemisphere). The bar graph illustrates how the main effects of group were present in both individual scan sessions (i.e., no interaction effect with scan session) for the left fusiform gyrus cluster

The TDC group showed significantly greater relative perfusion than the ASD group in four key clusters, which include the bilateral fusiform gyrus and the inferior temporal gyrus (see Fig. 1; Table 2). There was no evidence of greater relative perfusion in the ASD group compared to the TDC group.

**Table 2:** Group differences in rCBF

Peak voxel label (Harvard-Oxford Cortical Atlas)	Peak MNI coordinate			Voxels	Peak *t* score
	*X Y Z*				
*TDC > ASD*					
Left fusiform	− 32	− 14	− 34	317	4.15
Right inferior temporal gyrus	44	− 14	− 36	53	3.83
Left fusiform	− 30	0	− 40	7	3.08
Right fusiform	40	− 28	− 26	7	3.60

### Acceptable reliability of pCASL

ICCs ranged from good (0.50 < ICC < 0.75) to excellent (0.75 < ICC < 0.90) across both groups. See Table 3 for details.

**Table 3:** Intra-class correlations (ICC) for each cortical region by diagnostic group

Cortical region	ASD ICC [95% CI]	TDC ICC [95% CI]
Frontal	0.752 [0.748, 0.757]	0.746 [0.739, 0.748]
Insula	0.780 [0.769, 0.792]	0.764 [0.751, 0.775]
Occipital	0.729 [0.722, 0.736]	0.751 [0.744, 0.758]
Parietal	0.787 [0.783, 0.792]	0.776 [0.772, 0.781]
Temporal	0.711 [0.704, 0.718]	0.698 [0.691, 0.706]

### Correlation with behavioral metrics show perfusion relationships with face recognition performance

**Fig. 2:**
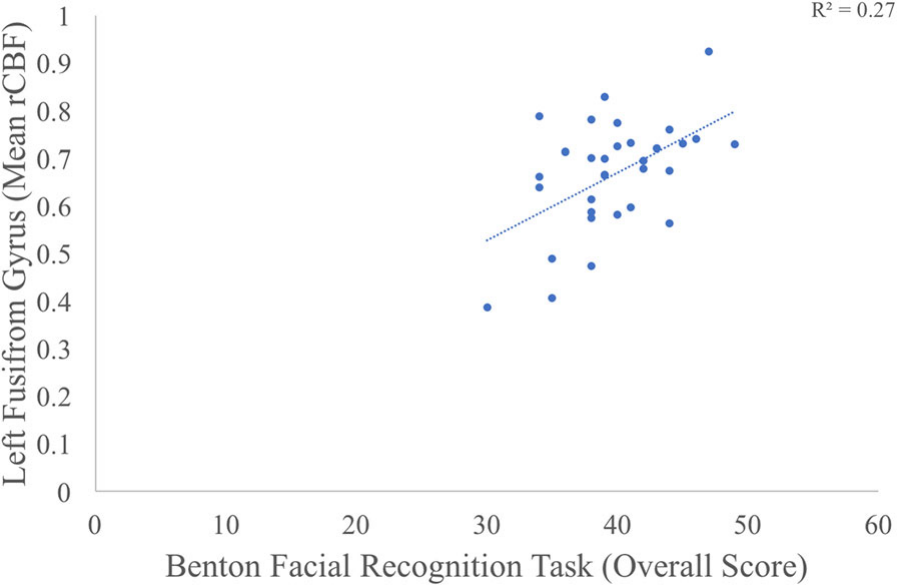
This scatterplot highlights the correlation between rCBF in the left fusiform gyrus and child performance on the Benton Facial Recognition Task

We correlated mean rCBF values within the four clusters that differed between groups with clinician ratings of ASD symptoms, parent ratings of socialization skills, and performance on a face recognition task. There was a moderate relationship between the largest cluster in the left fusiform gyrus and performance on the Benton Facial Recognition Test (*r* = 0.52, *p* < 0.05 (FDR-adjusted), [0.20, 0.73]; see Fig. 2). Partial correlations remained significant when removing variability related to age (*r* = 0.46, *p <* 0.05, FDR-adjusted) and FSIQ (*r* = 0.50, *p* < 0.05, FDR-adjusted). All other correlations were non-significant (all *r*s < |0.24|, *p*s > 0.19).

### Secondary analyses confirm primary analysis and yield no additional results

Secondary analyses tested for group differences in rCBF in the temporal lobe after removing the child with ASD and an IQ of 47. Of the four significant clusters identified in the primary analysis, only the largest cluster in the left fusiform gyrus remained significant, as did its correlation with the Benton Facial Recognition task (*r* = 0.51, *p* < 0.005). Additional analyses examining rCBF and absolute CBF across the entire brain, as well as within a frontal lobe gray matter mask yielded no significant group differences.

## Discussion

Children with ASD demonstrated reductions in rCBF in the bilateral fusiform gyrus and right inferior temporal lobe regions compared to controls—areas critical for social perception and cognition—while watching a video of natural scenes. These findings replicate the fusiform hypoactivation literature [30, 31, 37] but using ASL rather than BOLD fMRI. Furthermore, individual differences in rCBF of the left fusiform gyrus—the cluster with the greatest spatial extent—were associated with facial recognition performance, but not ASD symptoms. While findings of the left (not the right) fusiform gyrus being specifically correlated with face identity recognition skills was a bit surprising, bilateral activation during fMRI social perceptual tasks is the norm. Correlations between the left fusiform’s rCBF and the Benton Facial Recognition Test and not ASD symptom severity is unsurprising as the performance-based measure of facial recognition is more tightly coupled to the putative role of the fusiform gyrus than a broad stroke measure of ASD symptoms [59], perhaps due to the increased granularity and better psychometrics for the Benton Facial Recognition Test vs. the ADOS or the SRS-2.

This study is the first to evaluate the reliability of rCBF in people with ASD using pseudocontinuous ASL; over the same time period as most clinical trials, we observed moderate-to-good reliability for perfusion across gray matter cortex in both ASD and TDC groups. The present study moves us closer to identifying a biologically based marker of ASD that relates to a basic social perception deficit, while highlighting the value of ASL neuroimaging to the field of ASD. These results need to be replicated, but suggest that ASL could provide useful biological markers of ASD in a manner that could also be useful as an outcome measure for intervention trials.

The temporal lobe, and the fusiform gyrus more specifically, has been implicated in the core social and communication impairments in individuals with ASD [30, 31, 37]. In particular, reduced activation of the fusiform during the processing of faces has led to hypotheses focusing on how social information may be de-prioritized in favor of non-social information [29, 34, 60–63]. However, despite this body of work on the role of the fusiform gyrus in ASD symptoms, there have been numerous negative findings [64–69] raising concern about the reliability of this brain difference.

The present study represents a first step in addressing some of these existing concerns. It directly addresses the issue of reliability by demonstrating moderate to good reliability with ICCs across an ~ 8-week interval, and a lack of interaction effects between diagnostic group and session. With a growing emphasis on reproducibility in neuroscience research [70], the demonstration of reliability of CBF across the cortex in both samples is an important foundational step, and it aligns with other efforts to demonstrate reproducibility in functional connectivity (e.g., [71]). The stability of the group difference over time, suggests that measuring rCBF in the fusiform may be a reasonable biologically based marker for clinical trials aiming to improve social perception. However, additional work is needed to identify normative trajectories of rCBF across age, sex, and IQ so that we may quantify when ‘atypical’ levels have been reached at the individual level before this measure could be deemed an appropriate outcome for clinical trial use.

The present study also showed that rCBF has a dimensional relationship with facial recognition performance, such that greater perfusion in children with ASD is associated with better performance on a facial recognition task. This type of effect provides convergent evidence regarding the fusiform’s role in impairments seen in the ASD phenotype from an unrelated MRI modality (ASL vs. blood-oxygen-dependent-level) that has a stronger relationship to neural activity than other functional MRI sequences like BOLD fMRI.

To the best of our knowledge there is only one prior study evaluating rCBF in ASD using the ASL method, which is also the lone study failing to report hypoperfusion in ASD [9]. In Jann et al. (2015), hyperperfusion was observed in inferior temporal regions for the ASD group relative to controls, and no hypoperfusion was observed for the ASD group. There are methodological differences that may account for these discrepancies between the studies. One is that our study had children engage in a passive viewing task that included biological motion (animal movements), whereas the prior study had children rest with no visual stimulation. The second is that the prior study used a different ASL implementation that may have provided lower effective resolution despite identical nominal resolution. In particular, a pure-resting state with limited external stimulation may have influenced the degree of temporal cortex engagement for controls more than the ASD group. While speculative, there is some evidence that employing social stimuli built around context, narrative, or familiarity are much better at eliciting activation in temporal regions [27]. Indirect evidence of this possible explanation comes from a prior study of temporal lobe pathology in patients with mild cognitive impairment. These patients showed enhanced differences in temporal lobe CBF compared to controls during a memory task [26]. Future studies may seek to examine whether the presence and absence of complex, biologically meaningful information alters perfusion in temporal regions during ASL scans to a greater degree for typically developing children or neurotypical adults over those with an ASD diagnosis.

The present study has some notable limitations. This study does not include females, and so our findings of perfusion differences in the fusiform may be subject to interactions with biological sex. Future studies should directly target the inclusion of females to specify rCBF relative to females without ASD and males with ASD. This will inform whether the fusiform is a general marker or one specific to males. While children were instructed to watch the video, we did not monitor eye movements to confirm compliance. Future investigations should consider monitoring engagement with videos to determine if this influences results and also as a potential criterion for inclusion in analyses. This study had a wider IQ range in the ASD group than the TDC group leading to a significant difference with a large effect. Of note, while the ASD group continued to show lower relative perfusion in the largest cluster in the left fusiform gyrus relative to controls, the other three clusters in the temporal lobes were no longer significant. This pattern of finding suggests that lower IQ may be associated with some degree with lower relative perfusion in the temporal lobes. However, the robustness of group differences in the left fusiform gyrus and the correlation between relative perfusion in this region and face recognition skills, suggests the findings may be specific to social functioning and not merely an indicator of cognitive impairment. Furthermore, our study offers optimism that ASL could be implemented to identify relevant biomarkers for individuals with co-occurring ASD and intellectual disability diagnoses.

## Conclusions

Identification of a reliable biological marker in ASD remains elusive. The present study built upon existing functional MRI studies that have implicated the fusiform gyrus in the face processing impairments that are characteristic of the ASD phenotype. The present study measured rCBF using a non-invasive method, pseudocontinuous ASL. The results confirm early reports of hypoperfusion in the fusiform gyrus of individuals with ASD, and that greater perfusion in ASD is also associated with better performance on a face recognition task. Further, ASL was shown to have reasonably good reliability across an ~ 8-week period, which is notable since this time period corresponds with that of an average clinical trial. Thus, the present study has extended prior work in the fusiform in ASD by demonstrating its reliability and sensitivity to individual differences in ASD using an underutilized metric of neural function. This study has also provided the preliminary psychometric data needed for pursuing perfusion in the fusiform gyrus as a potential target in treatment studies.

## Abbreviations

ADOS: Autism Diagnostic Observation Schedule
ASD: Autism spectrum disorder
ASL: Arterial spin labeling
CBF: Cerebral blood flow
CSF: Cerebrospinal fluid
DAS-II: Differential Ability Scales, second edition
FAST: FMRIB’s Automated Segmentation Tool
FOV: Field of view
FSL: FMRIB Software Library
GM: Gray matter
ICC: Intra-class correlations
MNI: Montreal Neurological Institute
rCBF: Relative cerebral blood flow
RF: Radio frequency
ROI: Region of interest
SNR: Signal-to-noise ratio
SRS-2: Social Responsiveness Scale, second edition
TDC: Typically developing control
TFCE: Threshold-free cluster enhancement
TR/TE: Repetition time/echo time
VABS-II: Vineland Adaptive Behavior Scales, second edition
WM: White matter

## References

[1] Ohnishi T, Matsuda H, Hashimoto T, Kunihiro T, Nishikawa M, Uema T (2000). Abnormal regional cerebral blood flow in childhood autism. Brain.

[2] Zilbovicius M, Boddaert N, Belin P, Poline J-B, Remy P, Mangin J-F (2000). Temporal lobe dysfunction in childhood autism: a PET study. Am J Psychiatry.

[3] Burroni L, Orsi A, Monti L, Hayek Y, Rocchi R, Vattimo AG (2008). Regional cerebral blood flow in childhood autism: a Spet study with Spm evaluation. Nucl Med Commun.

[4] Gendry Meresse I, Zilbovicius M, Boddaert N, Robel L, Philippe A, Sfaello I (2005). Autism severity and temporal lobe functional abnormalities. Ann Neurol.

[5] Critchley HD, Daly EM, Bullmore ET, Williams SCR, Van Amelsvoort T, Robertson DM (2000). The functional neuroanatomy of social behaviourChanges in cerebral blood flow when people with autistic disorder process facial expressions. Brain.

[6] Starkstein SE, Vazquez S, Vrancic D, Nanclares V, Manes F, Piven J (2000). SPECT findings in mentally retarded autistic individuals. J Neuropsychiatry Clin Neurosci.

[7] George MSMD, Costa DCMD, Kouris K, Ring HAMRCP, Ell PJMD (1992). Cerebral blood flow abnormalities in adults with infantile autism. J Nerv.

[8] Yang WH, Jing J, Cheng MH, Wang X, Bao P, Wang QX (2011). Regional cerebral blood flow in children with autism spectrum disorders: a quantitative ^99^mTc-ECD brain SPECT study with statistical parametric mapping evaluation. Chin Med J Engl.

[9] Jann Kay, Hernandez Leanna M., Beck-Pancer Devora, McCarron Rosemary, Smith Robert X., Dapretto Mirella, Wang Danny J. J. (2015). Altered resting perfusion and functional connectivity of default mode network in youth with autism spectrum disorder. Brain and Behavior.

[10] Lassen NA, Ingvar DH, Skinhøj E (1978). Brain function and blood flow. Sci Am.

[11] Sokoloff L (1981). Localization of functional activity in the central nervous system by measurement of glucose utilization with radioactive deoxyglucose. J Cereb Blood Flow Metab.

[12] Tak S, Wang DJ, Polimeni JR, Yan L, Chen JJ (2014). Dynamic and static contributions of the cerebrovasculature to the resting-state BOLD signal. NeuroImage.

[13] Biswal BB, Kylen JV, Hyde JS (1997). Simultaneous assessment of flow and BOLD signals in resting-state functional connectivity maps. NMR Biomed.

[14] Chuang K-H, van Gelderen P, Merkle H, Bodurka J, Ikonomidou VN, Koretsky AP (2008). Mapping resting-state functional connectivity using perfusion MRI. NeuroImage.

[15] Fukunaga M, Horovitz SG, de Zwart JA, van Gelderen P, Balkin TJ, Braun AR (2008). Metabolic origin of bold signal fluctuations in the absence of stimuli Journal of Cerebral Blood Flow &amp. Metabolism J Cereb Blood Flow Metab.

[16] Liang X, Zou Q, He Y, Yang Y (2013). Coupling of functional connectivity and regional cerebral blood flow reveals a physiological basis for network hubs of the human brain. Proc Natl Acad Sci.

[17] Viviani R, Messina I, Walter M (2011). Resting state functional connectivity in perfusion imaging: correlation maps with BOLD connectivity and resting state perfusion. PLoS One.

[18] Zou Q, Wu CW, Stein EA, Zang Y, Yang Y (2009). Static and dynamic characteristics of cerebral blood flow during the resting state. NeuroImage.

[19] Williams DS, Detre JA, Leigh JS, Koretsky AP (1992). Magnetic resonance imaging of perfusion using spin inversion of arterial water. Proc Natl Acad Sci.

[20] Chiron C, Raynaud C, Mazière B, Zilbovicius M, Laflamme L, Masure MC (1992). Changes in regional cerebral blood flow during brain maturation in children and adolescents. J Nucl Med Off Publ Soc Nucl Med.

[21] Wang J, Licht DJ, Jahng G-H, Liu C-S, Rubin JT, Haselgrove J (2003). Pediatric perfusion imaging using pulsed arterial spin labeling. J Magn Reson Imaging JMRI.

[22] Wang J, Licht DJ (2006). Pediatric perfusion MR imaging using arterial spin labeling. Neuroimaging Clin N Am.

[23] Aguirre GK, Detre JA, Zarahn E, Alsop DC (2002). Experimental design and the relative sensitivity of BOLD and perfusion fMRI. NeuroImage.

[24] Wang J, Aguirre GK, Kimberg DY, Roc AC, Li L, Detre JA (2003). Arterial spin labeling perfusion fMRI with very low task frequency. Magn Reson Med.

[25] Rao H, Wang J, Tang K, Pan W, Detre JA (2007). Imaging brain activity during natural vision using CASL perfusion fMRI. Hum Brain Mapp.

[26] Xie L, Dolui S, Das SR, Stockbower GE, Daffner M, Rao H (2016). A brain stress test: cerebral perfusion during memory encoding in mild cognitive impairment. NeuroImage Clin.

[27] Olson IR, Plotzker A, Ezzyat Y (2007). The enigmatic temporal pole: a review of findings on social and emotional processing. Brain J Neurol.

[28] Pascual B, Masdeu JC, Hollenbeck M, Makris N, Insausti R, Ding S-L (2015). Large-scale brain networks of the human left temporal pole: a functional connectivity MRI study. Cereb Cortex.

[29] Schultz RT (2005). Developmental deficits in social perception in autism: the role of the amygdala and fusiform face area. Int J Dev Neurosci.

[30] Di Martino A, Ross K, Uddin LQ, Sklar AB, Castellanos FX, Milham MP (2009). Functional brain correlates of social and nonsocial processes in autism spectrum disorders: an activation likelihood estimation meta-analysis. Biol Psychiatry.

[31] Patriquin MA, DeRamus T, Libero LE, Laird A, Kana RK (2016). Neuroanatomical and neurofunctional markers of social cognition in autism spectrum disorder. Hum Brain Mapp.

[32] Nomi JS, Uddin LQ (2015). Face processing in autism spectrum disorders: from brain regions to brain networks. Neuropsychologia.

[33] Pelphrey KA, Shultz S, Hudac CM, Vander Wyk BC (2011). Research review: constraining heterogeneity: the social brain and its development in autism spectrum disorder. J Child Psychol Psychiatry.

[34] Dawson G, Webb SJ, McPartland J (2005). Understanding the nature of face processing impairment in autism: insights from behavioral and electrophysiological studies. Dev Neuropsychol.

[35] Harms MB, Martin A, Wallace GL (2010). Facial emotion recognition in autism spectrum disorders: a review of behavioral and neuroimaging studies. Neuropsychol Rev.

[36] van Rooij D, Anagnostou E, Arango C, Auzias G, Behrmann M, Busatto GF, et al. Cortical and Subcortical Brain Morphometry Differences Between Patients With Autism Spectrum Disorder and Healthy Individuals Across the Lifespan: Results From the ENIGMA ASD Working Group. Am J Psychiatry. 2017;175:359-69. 10.1176/appi.ajp.2017.17010100 PMC6546164 29145754

[37] Schultz RT, Gauthier I, Klin A, Fulbright RK, Anderson AW, Volkmar F (2000). Abnormal ventral temporal cortical activity during face discrimination among individuals with autism and Asperger syndrome. Arch Gen Psychiatry.

[38] American Psychiatric Association, DSM-5 Task Force (2013). Diagnostic and statistical manual of mental disorders: DSM-5.

[39] American Psychiatric Association (2000). Diagnostic and statistical manual of mental disorders DSM-IV-TR fourth edition.

[40] Gotham K, Risi S, Pickles A, Lord C (2007). The autism diagnostic observation schedule: revised algorithms for improved diagnostic validity. J Autism Dev Disord.

[41] Lord C, Rutter M, Le Couteur A (1994). Autism diagnostic interview-revised: a revised version of a diagnostic interview for caregivers of individuals with possible pervasive developmental disorders. J Autism Dev Disord.

[42] Gadow KD, Sprafkin J (2010). Child & Adolescent Symptom Inventory - fourth edition revised.

[43] Elliott CD (2007). Differential ability scales-II (DAS-II).

[44] Constantino JN, Gruber CP (2013). Social responsiveness scale, second edition (SRS-2).

[45] Sparrow SS, Cicchetti DV, Balla DA (2005). Vineland adaptive behavior scales, second edition (Vineland-II).

[46] Benton AL, Van Allen MW (1968). Impairment in facial recognition in patients with cerebral disease. Cortex.

[47] Jain V, Duda J, Avants B, Giannetta M, Xie SX, Roberts T (2012). Longitudinal reproducibility and accuracy of pseudo-continuous arterial spin-labeled perfusion MR imaging in typically developing children. Radiology.

[48] Jenkinson M, Beckmann CF, TEJ B, Woolrich MW, Smith SM (2012). FSL. NeuroImage.

[49] Wang Z, Aguirre GK, Rao H, Wang J, Fernández-Seara MA, Childress AR (2008). Empirical optimization of ASL data analysis using an ASL data processing toolbox: ASLtbx. Magn Reson Imaging.

[50] Wang J, Alsop DC, Song HK, Maldjian JA, Tang K, Salvucci AE (2003). Arterial transit time imaging with flow encoding arterial spin tagging (FEAST). Magn Reson Med.

[51] Jenkinson M, Bannister P, Brady M, Smith S (2002). Improved optimization for the robust and accurate linear registration and motion correction of brain images. NeuroImage.

[52] Woolrich M (2008). Robust group analysis using outlier inference. NeuroImage.

[53] Woolrich MW, Jbabdi S, Patenaude B, Chappell M, Makni S, Behrens T (2009). Bayesian analysis of neuroimaging data in FSL. NeuroImage.

[54] Hayasaka S, Nichols TE (2003). Validating cluster size inference: random field and permutation methods. NeuroImage.

[55] Nichols TE, Holmes AP (2002). Nonparametric permutation tests for functional neuroimaging: a primer with examples. Hum Brain Mapp.

[56] Zhang Y, Brady M, Smith S (2001). Segmentation of brain MR images through a hidden Markov random field model and the expectation-maximization algorithm. IEEE Trans Med Imaging.

[57] R Core Team. R: A language and environment for statistical computing. [Internet]. Vienna: R Foundation for Statistical Computing; 2014. Available from: http://www.r-project.org/.

[58] Revelle W. Psych: procedures for personality and psychological research [internet]. Evanston: Northwestern University; 2015. Available from: http://cran.r-project.org/package=psych.

[59] Abbott AE, Nair A, Keown CL, Datko M, Jahedi A, Fishman I (2016). Patterns of atypical functional connectivity and behavioral links in autism differ between default, salience, and executive networks. Cereb Cortex.

[60] Grelotti DJ, Klin AJ, Gauthier I, Skudlarski P, Cohen DJ, Gore JC (2005). fMRI activation of the fusiform gyrus and amygdala to cartoon characters but not to faces in a boy with autism. Neuropsychologia.

[61] Schultz RT, Grelotti DJ, Klin A, Kleinman J, Van der Gaag C, Marois R (2003). The role of the fusiform face area in social cognition: implications for the pathobiology of autism. Philos Trans R Soc Lond Ser B Biol Sci.

[62] Kohls G, Yerys BE, Schultz RT (2014). Striatal development in autism: repetitive behaviors and the reward circuitry. Biol Psychiatry.

[63] Kohls G, Chevallier C, Troiani V, Schultz RT (2012). Social “wanting” dysfunction in autism: neurobiological underpinnings and treatment implications. J Neurodev Disord.

[64] Hadjikhani N, Joseph RM, Snyder J, Chabris CF, Clark J, Steele S (2004). Activation of the fusiform gyrus when individuals with autism spectrum disorder view faces. NeuroImage.

[65] Hadjikhani N, Joseph RM, Snyder J, Tager-Flusberg H (2007). Abnormal activation of the social brain during face perception in autism. Hum Brain Mapp.

[66] Bookheimer SY, Wang AT, Scott A, Sigman M, Dapretto M (2008). Frontal contributions to face processing differences in autism: evidence from fMRI of inverted face processing. J Int Neuropsychol Soc.

[67] Dalton KM, Nacewicz BM, Alexander AL, Davidson RJ (2007). Gaze-fixation, brain activation, and amygdala volume in unaffected siblings of individuals with autism. Biol Psychiatry.

[68] Pierce K, Müller RA, Ambrose J, Allen G, Courchesne E (2001). Face processing occurs outside the fusiform “face area” in autism: evidence from functional MRI. Brain.

[69] Pierce K, Haist F, Sedaghat F, Courchesne E (2004). The brain response to personally familiar faces in autism: findings of fusiform activity and beyond. Brain.

[70] Button KS, Ioannidis JPA, Mokrysz C, Nosek BA, Flint J, Robinson ESJ (2013). Power failure: why small sample size undermines the reliability of neuroscience. Nat Rev Neurosci.

[71] Supekar K, Uddin LQ, Khouzam A, Phillips J, Gaillard WD, Kenworthy LE (2013). Brain Hyperconnectivity in children with autism and its links to social deficits. Cell Rep.

